# Estimating SARS Incubation Period

**DOI:** 10.3201/eid1008.040284

**Published:** 2004-08

**Authors:** Tze-wai Wong, Wilson Tam

**Affiliations:** * The Chinese University of Hong Kong, Hong Kong

**Keywords:** incubation period, SARS, multiple contacts

**To the Editor:** In a recent article, Meltzer described a simulation method to estimate the incubation period for patients infected with SARS with multiple contact dates (*1*). In brief, he assumed a uniform distribution of all possible incubation periods derived from these contact dates for each patient and randomly selected an incubation period from all contact dates for each patient to obtain a distribution of the incubation period for all 19 patients. The process is repeated 10,000 times to obtain an overall frequency distribution of the incubation period.

Instead of using this cumbersome iterative approach, the same results can be obtained by a simple method. When a uniform distribution is assumed for all possible incubation periods, the expected frequency for a day *x* as the incubation period is either 0 or 1/(total number of possible days). Taking the first patient (Canada 1) in (1) as an example, the expected frequency for 1, 2, 3, . . . , 18 days is 0, 1/11, 1/11, 1/11, 1/11, 1/11, 1/11, 1/11, 1/11, 1/11, 1/11, 1/11, 0, 0, . . . , 0. The expected frequencies for the other patients are available in the Table.

**Table:** Expected frequency distribution of incubation period for SARS patient with multiple contacts

Patients^a^	Days																	
	1	2	3	4	5	6	7	8	9	10	11	12	13	14	15	16	17	18
Ca1	0	1/11	1/11	1/11	1/11	1/11	1/11	1/11	1/11	1/11	1/11	1/11	0	0	0	0	0	0
Ca2	1/4	1/4	1/4	1/4	0	0	0	0	0	0	0	0	0	0	0	0	0	0
Ca3	1/2	0	0	1/2	0	0	0	0	0	0	0	0	0	0	0	0	0	0
Ca4	1/11	1/11	1/11	1/11	1/11	1/11	1/11	1/11	1/11	1/11	1/11	0	0	0	0	0	0	0
Ca5	1/14	1/14	1/14	1/14	1/14	1/14	1/14	1/14	1/14	1/14	1/14	1/14	1/14	1/14	0	0	0	0
Ca7	0	0	1/2	0	0	0	0	0	0	1/2	0	0	0	0	0	0	0	0
Ca8	0	0	1	0	0	0	0	0	0	0	0	0	0	0	0	0	0	0
Ca10	1/6	1/6	1/6	1/6	1/6	1/6	0	0	0	0	0	0	0	0	0	0	0	0
HK2	0	1	0	0	0	0	0	0	0	0	0	0	0	0	0	0	0	0
HK3	0	1	0	0	0	0	0	0	0	0	0	0	0	0	0	0	0	0
HK4	0	0	0	0	0	1	0	0	0	0	0	0	0	0	0	0	0	0
HK5	0	1	0	0	0	0	0	0	0	0	0	0	0	0	0	0	0	0
HK6	1/6	1/6	1/6	1/6	1/6	1/6	1/6	0	0	0	0	0	0	0	0	0	0	0
HK7	0	0	0	0	1/7	1/7	1/7	1/7	1/7	1/7	1/7	0	0	0	0	0	0	0
HK8	0	0	0	0	1/7	1/7	1/7	1/7	1/7	1/7	1/7	0	0	0	0	0	0	0
HK9	1/5	1/5	1/5	1/5	1/5	0	0	0	0	0	0	0	0	0	0	0	0	0
HK10	0	1/6	1/6	1/6	1/6	1/6	1/6	0	0	0	0	0	0	0	0	0	0	0
US1	0	0	0	0	0	1/7	0	0	0	0	0	0	1/7	1/7	1/7	1/7	1/7	1/7
US2	0	0	0	0	0	0	1/6	1/6	1/6	1/6	1/6	1/6	0	0	0	0	0	0
Tot. exp. freq.	1.45	4.40	2.70	1.70	1.24	2.18	0.87	0.71	0.71	1.21	0.71	0.33	0.21	0.21	0.14	0.14	0.14	0.14

^a^Ca, Canada; HK, Hong Kong; US, United States

The total expected frequency for each day is the sum of the expected frequencies for all patients for that day. Therefore, the frequency distribution of the incubation period is given by dividing each total expected frequency by the sum of the total expected frequencies (x 100%) and is 7.6, 22.1, 14.2, 9.0, 6.5, 11.5, 4.6, 3.7, 3.7, 6.4, 3.7, 1.7, 1.1, 1.1, 0.7, 0.7, 0.7, 0.7. This is identical to the frequency distribution shown in Figure 1 of the paper by Meltzer (*1*).
