# Supplementary material for: Estimating SARS Incubation Period
Source: Emerg Infect Dis. 2004 Aug;10(8):1503–4. doi: 10.3201/eid1008.040284 (PMC3320416; doi:10.3201/eid1008.040284)
Supplement: Related texts

# Reply: Estimating SARS Incubation Period

**DOI:** 10.3201/eid1008.040427

**Authors:** Martin I. Meltzer

**Affiliations:** * Centers for Disease Control and Prevention, Atlanta, Georgia, USA

**In Reply:** Drs. Wong and Tam (1) are correct in stating that their method of calculating mean frequencies of possible incubation periods for patients with severe acute respiratory syndrome (SARS) is simpler than the method that I presented (2). However, their method cannot replicate the confidence intervals shown in Figure 1 in my article. Their suggested methodology can only replicate Figure 2 in my article, which shows the cumulative distribution of the mean frequencies of individual incubation periods.

The comparative complexity of my method provides data that are essential for making public health decisions. For example, public health officials need to know incubation periods to determine appropriate periods of quarantine and isolation and how long to conduct intensive (and expensive) surveillance after the last clinical case has been reported. To reduce costs and to enhance public support, public health officials may keep quarantine and isolation periods to a minimum. They also need to know the risk for failure of such interventions attributable to patients with relatively long incubation periods. Both Figure 2 in my article and Drs. Wong and Tam's data show that approximately 95% of the mean incubation period will be <12 days (i.e., 5% will incubate for 13 to 18 days). By summing the 95th percentiles for days 13 through 18 from my Figure 1 , it can be seen that there is a probability that <30% of patients will have incubation periods >12 days (the actual probability of any given percentage incubating for >12 days can be easily calculated by using the spreadsheet which is a Technical Appendix to my article). Public health officials need to understand the degree of variability associated with any data used to make public health policies. Sole reliance on the mean incubation periods (or mean frequencies) will hide more than is shown, which increases the probability of failed public health interventions.

## Supplementary Material

Technical Appendix Open reading frames of African swine fever virus.

## References

[1] Wong TW, Tam W. Estimating SARS incubation period [letter]. Emerg Infect Dis. 2004;10:1503–4.1550339710.3201/eid1008.040284PMC3320416

[2] Meltzer MI. Multiple contact dates and SARS incubation periods. Emerg Infect Dis. 2004;10:207–9.1503068410.3201/eid1002.030426PMC3322923
